# Liver injury in COVID-19: clinical features and treatment management

**DOI:** 10.1186/s12985-021-01593-1

**Published:** 2021-06-09

**Authors:** Dongdong Yu, Qingru Du, Shengguang Yan, Xu-Guang Guo, Yehao He, Guodong Zhu, Kewei Zhao, Shi Ouyang

**Affiliations:** 1 Department of Traditional Chinese Medicine, The Fifth Affiliated Hospital of Guangzhou Medical University, Guangzhou, 510150 China; 2 Department of Infection Disease, The Fifth Affiliated Hospital of Guangzhou Medical University Guangzhou, Guangzhou, 510150 China; 3 School of Public Health, North China University of Science and Technology, Tangshan, 063210 Hebei China; 4 Department of Clinical Laboratory Medicine, The Third Affiliated Hospital of Guangzhou Medical University, Guangzhou, 510150 China; 5 Department of Clinical Laboratory Medicine, The Fifth Affiliated Hospital of Guangzhou Medical University, Guangzhou, 510150 China; 6 Departments of Geriatrics and Oncology, Guangzhou First People’s Hospital, Guangzhou, 510180 China; 7 School of Medicine, South China University of Technology, Guangzhou, Guangdong China; 8 The Third Affiliated Hospital of Guangzhou University of Chinese Medicine, Guangzhou, 510378 Guangdong China; 9 Department of Preventive Medicine, School of Public Health, Guangzhou Medical University, Guangzhou, 510150 China

**Keywords:** COVID-19, SARS-CoV2, Liver injury

## Abstract

Severe acute respiratory syndrome coronavirus-2 (SARS-CoV-2) has spread to many countries around the world. In addition to lung disease, severe cases also displayed varying degrees of liver injury. This article will describe the latest developments regarding coronavirus and the pathogenesis of liver injury, the prone population and clinical characteristics of these patients, as well as providing some suggestions for clinical treatment.

## Background

Since December 2019, severe acute respiratory syndrome coronavirus-2(SARS-CoV-2), named by the International Classification Committee of viruses, has brought new health threats and challenges to the world. In March 11, 2020, the coronavirus disease 2019(COVID-19) was declared a global pandemic by WHO. As of March 8, 2021, there have been 156,496,592 confirmed cases of COVID-19 worldwide, including 3,264,143 deaths, which were reported to WHO. Abnormal liver function is often found in patients with COVID-19, and some studies [1] have shown that SARS-COV-2 is associated with liver dysfunction or damage. In China, there are 300 million patients with chronic liver disease, so clinicians should be alert to the possibility of liver injury in COVID-19 patients.

## Occurrence of liver injury in COVID-19

### Incidence of liver injury

The largest cohort study in China [2] included 1,099 patients with COVID-19, of whom 21 (2.1%) had hepatitis B, 21.3% and 22.2% had elevated Alanine Amino Transaminase (ALT) and Aspartate Amino Transaminase (AST), and 10.5% had abnormal bilirubin. Cai [3] et al. analyzed 417 patients with COVID-19 in Shenzhen. The standards of abnormal liver function are: ALT > 40 U/L, AST > 40 U/L, GGT > 49 U/L, ALP > 135 U/L, and total bilirubin (TBIL) > 17.1 mmol/L [3]. COVID-19 associated hepatic injury should be defined as ALT or AST exceeding 3 times the upper limit of the normal value, and ALP, GGT or TBIL exceeding 2 times the upper limit of the normal value [3]. In order to further describe the liver injury, it is also classified as hepatocellular, cholestatic and mixed type. Patients with elevated ALT and/or AST more than 3 times the upper limit of normal (ULN) are classified as hepatocyte type; Patients with raised ALP or GGT twice the ULN are classified as cholestatic type, and mixed type patients have both (see Table 1). COVID-19 was classified into mild and severe cases according to National Health Commission of the People's Republic of China Handbook of Prevention and Treatment of the Pneumonia Caused by the Novel Coronavirus (2019-nCoV) (in Chinese) 2020 (see Table 1) [4]. The study found that 41.0% and 5.0% of the 417 patients had abnormal liver function test and liver injury at admission. However after 2 weeks of hospitalization, patients with abnormal liver function tests and liver injury increased to 76.3% and 21.5% respectively. Bloom [5] also observed the same trend of liver biochemical reaction in patients with COVID-19. The ratio of patients with at least one abnormal liver biochemical index increased from 69 to 93%. It can be seen that the incidence of abnormal liver function test gradually increased with the extension of observation time. Previous studies reported that the proportion of elevated ALT was 9.6–37.6% [3, 6–9], the proportion of elevated AST was 14.8–36% [3, 6–10], the proportion of abnormal GGT was 13.0–24.4% [3, 6], and the proportion of abnormal total bilirubin was 5.1–18% [2, 3, 7, 9]. Generally speaking, incidence of abnormal liver function examination in hospitalized patients with COVID-19 ranges from 10.5 to 69% [3, 5, 11–13]. Most studies have shown that abnormal liver function tests are mainly caused by elevated AST and ALT, and elevated AST was more common than ALT. Compared with AST and ALT, elevated GGT and total bilirubin are less common.

**Table 1:** The definition of some concepts in this paper

Classification	Definition
Liver injury [3]	ALT and/or AST over 3 × ULN, ALP, GGT, and/or TBIL over 2 × ULN
Hepatocellular [3]	elevated ALT and/or AST more than 3 × ULN
Cholestatic [3]	elevated ALP or GGT over twice the ULN
Mixed (Abnormality) [3]	Both elevated ALT and/or AST more than 3 × ULN, and elevated ALP or GGT over twice the ULN
Cytokine Storm	The inflammatory markers C-reactive protein (CRP), serum ferritin, LDH, D-dimer, IL-6 and IL-2 in severe COVID-19 patients are significantly increased
Mild cases of COVID-19 [4]	patient can present as common symptoms: fever, dry cough, fatigue, headache, sore throat
Severe cases of COVID-19 [4]	Patient who fits any one of the following condition: 1. Respiratory rate ≥ 30 breath/min 2. SpO_2_ ≤ 93% 3.PaO_2_/FiO_2_ ≤ 300 mmHg (1 mmHg = 0.133 kPa)

### Liver injury prone population

A summary of the liver injury prone population is given in Table 2, which is discussed in detail below. Several studies have reported that the severe cases of COVID-19 were more likely to have severe liver injury compared to mild cases [2, 13, 14]. Male patients were more likely to have liver function injury than female (P < 0.05) [7]. The analysis of 417 patients with COVID-19 in Shenzhen showed that the patients with abnormal liver function were older, had higher proportion of cough as the first symptom, higher BMI, greater proportion of male, had not contacted with SARS-CoV-2, and had more of underlying liver diseases(all P < 0.05) [3]. Some studies showed that there was no significant difference in age, medication history and symptoms between the two groups [7]. A multicenter study in USA found that the proportion of AST was more than 3 times higher than the upper limit of normal value in patients with underlying liver diseases than that in patients without underlying liver diseases. Other indicators such as increased GGT were more common in patients with underlying liver diseases, indicating that patients with underlying liver diseases (fatty liver or nonalcoholic fatty liver disease) were more likely to have abnormal liver function test than patients without underlying liver diseases [15]. Logistic regression analysis showed that the degree of lung lesions on CT was a predictor of liver dysfunction (P < 0.05). Severe lung lesions were more likely to cause liver dysfunction [6]. Another study [16] showed that among the COVID-19 patients who received organ transplantation, the ICU admission rate was as high as 33.3%, and 20% of patients died. It can be seen that the death risk of liver and kidney transplantation patients is increased.

**Table 2:** Liver injury prone population

Author	Liver injury prone population
Guan [2], Mao [13], Henry [14]	Severe COVID-19 patients > Mild COVID-19 patients
Xie [7]	Male > Female; The patients with severe lung disease; But patients’ age, previous medication history and symptoms without significant difference (P > 0.05)
Mao [13]	Hubei Province in China > other parts of China; Severe COVID-19 Patients > Mild COVID-19 patients
Cai [3]	Aged; Initial symptoms is cough; Higher BMI; Male; Patients with underlying liver diseases;
Zhang [6]	The patients with severe lung disease
Singh [15]	Patients with underlying liver diseases > Patients without underlying liver diseases

## Possible mechanism of liver injury induced by COVID-19

**Fig. 1:**
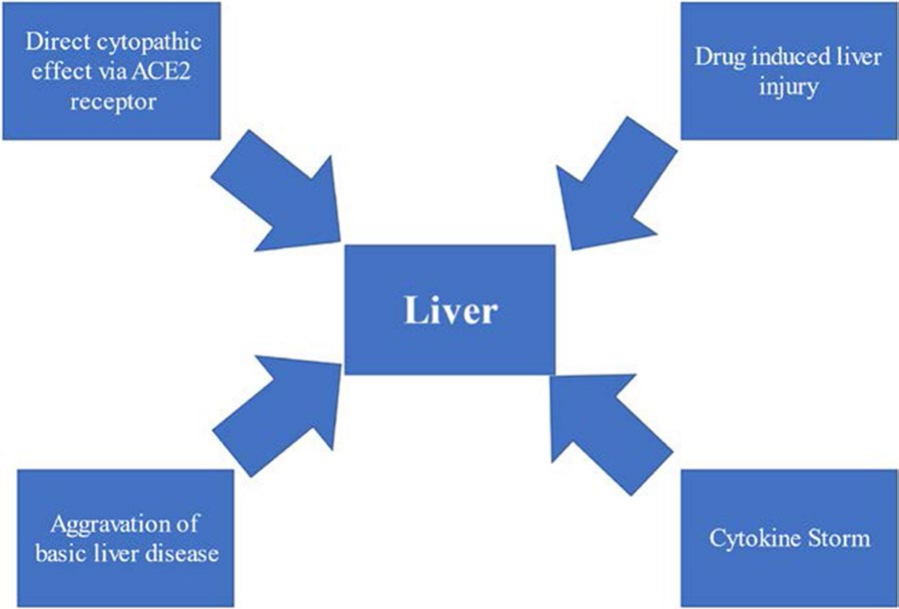
Possible mechanism of liver injury induced by COVID-19

Figure 1 summarizes the possible mechanisms of liver injury, which will be discussed below.

### Direct damage

Angiotensin-converting enzyme 2 (ACE2) is a key receptor of SARS-CoV-2 virus [17]. Xiaoqiang Chai et al. evaluated the cell-specific expression of the ACE2 receptor in healthy liver tissues and found that ACE2 was expressed in 2.6% of hepatocytes and 59.7% of cholangiocytes, suggesting that liver injury may be caused by direct viral invasion [18]. A recent study by Wu et al. found that half of COVID-19-infected patients who had completely cleared respiratory tract infections had virus shedding in their fecal specimens up to 11 days after viral detection in respiratory tract samples became negative [19]. This suggests that there may be viral replication in extrapulmonary sites (digestive tract, liver).

### Drug-induced liver injury

Drugs for COVID-19 may cause liver damage. Some of these drugs, including lopinavir/ritonavir, rececivir, chloroquine, tocilizumab, mitifovir and traditional Chinese medicine, may have hepatic toxicity in some patients [20]. Some researchers [21] analyzed 148 cases of COVID-19 confirmed in Shanghai. The results showed that there were no significant difference in the pre hospital medication between the patients with normal liver function and those with abnormal liver function. However, the utilization rate of lopinavir/ritonavir in patients with new liver injury after admission was significantly higher than that in patients with normal hepatic function. Therefore, off-target drug actions may be one of the causes of COVID-19-related liver injury. It is suggested that monitoring the changes of liver function and timing of medication in patients with COVID-19 during hospitalization can better judge the causal relationship between drugs and liver injury.

### Cytokine Storm

Immune mediated injury caused by severe inflammatory reaction after COVID-19 infection can also cause liver injury [22]. The inflammatory markers C-reactive protein (CRP), serum ferritin, LDH, D-dimer, IL-6 and IL-2 in severe COVID-19 patients are significantly increased leading to a “cytokine storm” (see Table 1). Cytokine storm often leads to sudden deterioration of the patient's condition as the patient quickly enters the state of multiple organ failure, and the systemic inflammation caused by it can lead to secondary liver injury [23].

### Aggravation of underlying liver diseases

The prevalence of chronic liver disease in patients with COVID-19 was reported in previous studies, and the overall prevalence was 2–11% [12, 24]. Studies have shown that obesity may aggravate the severity of COVID-19 patients. Patients with metabolically related fatty liver disease have additional metabolic risk factors, which may be associated with more severe disease phenotype [25]. It is not known whether patients with underlying liver disease are more likely to be infected with COVID-19, but Da [26] and others predicted that patients with alcohol use disorder (AUD) and alcohol-related liver disease (ALD) may be one of the most affected populations. The main reasons are that these patients cannot meet with doctors regularly, hospital resources transfer, social isolation, resulting in psychological decompensation, increased drinking or recurrence.

In addition, the authors suggest that liver damage may be caused by viral reactivation of existing liver diseases. Patients with chronic liver diseases are more likely to suffer from COVID-19 related liver damage. Some biological drugs such as tocilizumab (IL-6 receptor blockade) and baricitinib (janus kinase inhibitor, might interrupt endocytosis of the virus and intracellular assembly of virus particles) may also cause HBV activation, leading to deterioration of liver function [20]. At present, the exact cause of liver injury in patients with COVID-19 is still unclear. However, it has been observed that the increase of liver enzymes in patients with COVID-19 is mostly mild and lasts for a short time. It is speculated that systemic inflammatory reaction and drug-induced are more likely.

## Clinical characteristics of liver injury in COVID-19 patients

### Pathophysiological characteristics

Liver biopsy specimens from patients with COVID-19 showed moderate microvascular steatosis and mild lobular and portal vein activity [27]. Autopsy results of a dead COVID-19 patient showed mild infiltration of hepatic lobules, infiltration of small lymphocytes, sinusoidal expansion of central lobule, and patchy necrosis [28]. There was no obvious inflammatory cell infiltration around the portal vein and the terminal hepatic vein, which is basically consistent with the mode of acute liver injury.In other words, there is no cytoplasmic balloon like change, mallory glass like change, or extracellular fibrosis, and more serious histological changes such as obvious coagulation necrosis and severe cholestasis have not been seen [29].

### The characteristics, degree and time trend of liver enzyme elevation in patients with liver injury

**Fig. 2:**
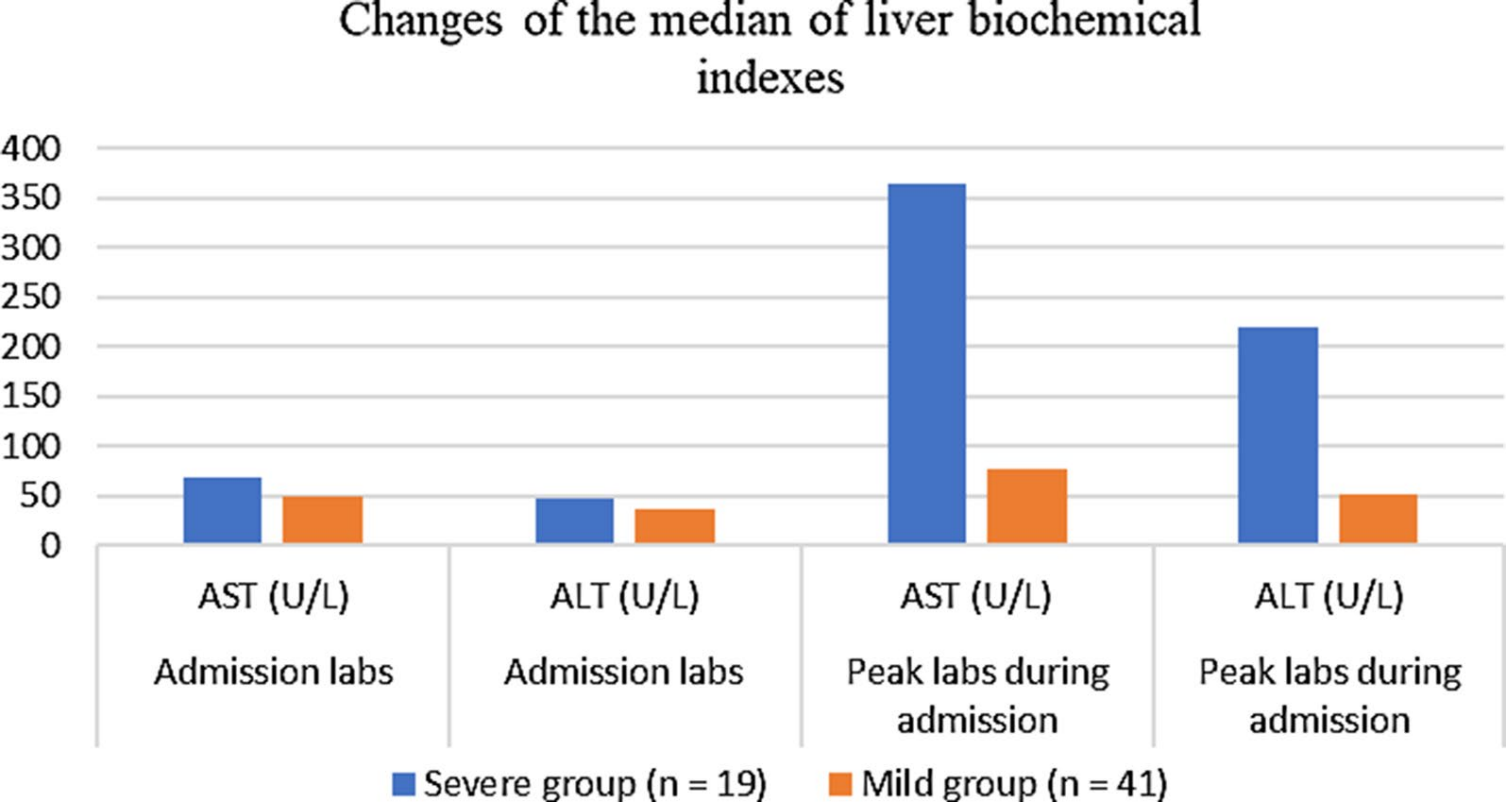
Change of the median of liver biochemical indexs

The situation of patients with liver injury from different studies are summarized in Table 3. which are detailed explanations of them. Among the 41 hospitalized patients first reported in Wuhan, China, 37% of them had elevated AST. The average levels of ALT, AST and total bilirubin were 32.0 (21.0–50.0) U/L, 34.0 (26.0–48.0) U/L, and 11.7 (9.5–13.9) μmol/L [10]. According to the biochemical analysis of 99 patients with COVID-19 infection in Wuhan area by Chen [9], the average level of ALT was 39.0 (22.0–53.0) U/L, and the average level of AST was 34.0 (26.0–48.0) U/L. The average level of total bilirubin was 15.1 (7.3) μmol/L, and liver enzymes were only slightly increased. Zhang [6] and others analyzed 115 cases of COVID-19 patients in Wuhan, and found that ALT was increased in 11 cases, and only 1 case was higher than 150u/L. In addition, AST in 17 cases was increased in the range of 40–120 U/L; TBIL in 7 cases was increased in the range of 21–31.5 μmol/L; ALP in 6 cases was increased ito 120–300 U/L; GGT in 15 patients was increased, and GGT in 3 patients exceeded 142.5 U/L. Wang et al. reported that the levels of ALT (35 vs 23, P = 0.007) and AST (52 vs 29, P < 0.001) in ICU patients were significantly higher than those in non ICU patients [30]. Filipe S. Cardoso reported the situation of liver injury in critically ill patients. During the 10 days of ICU observation, ALT and AST only slightly increased, and the highest value was no more than twice of the upper limit. However, with extended time, the increase of GGT became more and more obvious, reaching 3 times of the upper limit of normal value. The median peak value of GGT occurred 8 days after ICU admission. Generally speaking, liver injury was not serious, and late cholestasis was common [31]. Similar results were observed by Cai [3]. 417 patients with COVID-19 in Shenzhen were analyzed. It was found that most of the patients had abnormal liver test results within 1–2 × the upper limit of normal value, and only a few (< 4%) patients had abnormal liver test results higher than 2 × the upper normal value. GGT increased more significantly than other indicators, 12.71% of patients had 1–2 times the normal upper limit value, 1.2% of patients had 2–3 times the normal upper limit value, 10 cases of 2.4% patients had more than 3 times the normal upper limit values. Li [8] and others observed 25 fatal cases in patients infected with COVID-19. Among these patients, the average levels of ALT and AST were 24 (16.5–46) U/L and 37 (29.5–57.5) U/L, and the liver function was only slightly abnormal, but almost all patients had lower albumin (32.8 (28.6–36.0) μmol/L. Bloom [5] and others evaluated the change trend of ALT and AST with time in 60 patients with COVID-19, and found that the change trend of ALT and AST was basically the same. However with more time, it could slightly increase, reaching the peak on the 9th day of admission, and then decreasing slowly, with the increase range not more than 40 U/L. The comparison of the median of liver biochemical indexes between mild cases and severe cases at admission and at the peak of hospitalization is shown in Fig. 2. As can be seen from Fig. 2, in mild cases, the median changes of AST and ALT during admission and peak hospitalization were relatively small. In severe cases, AST and ALT at the peak of hospitalization were higher than those at admission. In addition, a retrospective analysis found that the percentage of mild cases in patients with elevated ALT and AST was 12.6%, while the percentage of severe cases was 46.2%. During the treatment period, 19% of the patients had elevated liver function parameters, but most of the patients had only mild and isolated elevation of ALT and AST, and most of the patients had normal liver indexes at discharge. However, patients with severe disease were more likely to have abnormal liver function [32]. Generally speaking [12], in mild cases of COVID-19, the increase of liver enzymes is usually mild, and severe liver damage is rare, lasting for a short time. Liver damage is usually transient and can be returned to normal without any special treatment. However, when severe liver damage occurs, hepatoprotective drugs are usually given to such patient, such as l-ornithine-l-aspartate [33].

**Table 3:** Notes on liver injury

Author	Patient	Abnormal liver biochemical indexes	Liver injury note	Time trend of liver enzyme elevation
Huang [10]	41	ALT: 32.0 (21.0–50.0) U/L AST: 34.0 (26.0–48.0) U/L TBIL: 11.7 (9.5–13.9) μmol/L	There were significant differences in liver biochemical indexes between severe patients and mild patients (all P < 0.05)	Not mentioned
Chen [9]	99	ALT: 39.0 (22.0–53.0) U/L AST: 34.0 (26.0–48.0) U/L TBIL: 15.1 (7.3) μmol/L	Most patients had mild liver dysfunction	Liver enzymes increased slightly
Zhang [6]	115	ALT: 25.71 (9–50) U/L AST: 28.30 (15–40) U/L TBIL: 11.31 (5–21) μmol/L	Slight abnormality of liver function; There were significant differences in liver biochemical indexes between severe patients and mild patients (all P < 0.05)	The changes of ALT, AST and TBIL in ICU patients were not significant with time (P > 0.05)
Bloom [5]	60	ALT: 39 (15.0–63.0) U/L AST: 55 (18–92) U/L TBIL: 0.9 ± 2.2 μmol/L	It has been proved that patients with severe COVID-19 have higher biochemical levels of liver	ALT and AST showed the same trend With the extension of time, there was a slight increase, which reached the peak on the ninth day, and then decreased slowly, and the increase was not more than 40 U/L
Wang [30]	138	ALT: 24 (16–40) U/L AST: 31 (24–51) U/L TBIL: 9.8 (8.4–14.1) μmol/L	The levels of ALT and AST in ICU patients were significantly higher than those not in ICU patients	Not mentioned
Cardoso [31]	20 (critically ill patients)	Not mentioned	Liver injury is common, but usually transient and not severe. However, cholestasis is obvious	ALT and AST only increased slightly(10d in ICU), and the highest < 2 × ULN GGT increased more and more obviously, reaching 3 × ULN
Cai [3]	417	ALT: 21 (15–31) U/L AST: 26.5 (21–35) U/L TBIL: 10.9 (8.3–16.3) μmol/L	AST, ALT and GGT were higher in severe patients	The increase of ALT and GGT was common, but the frequency of AST and TBIL was slightly lower
Xu [8]	25 (death)	ALT: 24 (16.5–46) U/L AST: 37 (29.5–57.5) U/L	Slight abnormality of liver function	Not mentioned
Wang [32]	105	ALT: 23.5 (14.0–36.0) U/L (N = 105) AST: 24.2 (19.7, 34.8) U/L (N = 50) TBIL: 10.2 (7.4, 12.9) μmol/L (N = 50)	Severe COVID-19 patients are more likely to have liver dysfunction	During the treatment, most of the patients only had mild or isolated elevation, and most of them were normal after discharge

## Effect of liver injury on prognosis of patients with COVID-19

The meta-analysis conducted by Henry [14] and others tried to find the blood, biochemical and immune biomarkers related to the severity and mortality of COVID-19. The results showed that the biomarkers of inflammation, myocardial injury, liver and kidney function and coagulation function in severe and critical patients were significantly higher than those in non-severe patients. It was suggested that liver injury may be related to severe COVID-19 infection. A retrospective analysis was conducted to compare the clinical characteristics of patients with and without liver function injury. The results showed that compared with patients without liver injury, the hospitalization time of patients with liver injury was significantly longer (P < 0.05) [7]. A multicenter retrospective cohort study found that abnormal AST was the most associated with the risk of death in patients with COVID-19 compared with other indicators of liver injury during hospitalization (HR 4.81–14.87, P < 0.001) [34]. In a multicenter study conducted by Singh [15], 2780 patients with COVID-19 were included. After adjusting for age, BMI, complications and other factors, patients with underlying liver disease had higher mortality (12% vs 4%, RR 3.0, 95% CI 1.5–6.0) and hospitalization rate (48% vs 36%, RR 1.3 (1.1,1.6). Compared to patients without NAFLD, patients with NAFLD had a higher risk of disease progression [6.6% (5/126) vs 44.7% (34/76), P < 0.001] [35]. However, Bolin Wang et al. conducted a meta-analysis on 1558 patients with COVID-19 and found that hypertension, diabetes mellitus, COPD, cardiovascular disease and cerebrovascular disease were the main risk factors for the severity of COVID-19 patients, while there was no correlation between liver disease, malignant tumor or kidney disease and disease severity [36]. The prediction model established by Gong [37] and others showed that elderly patients, high serum LDH, C-reactive protein, coefficient of variation of red blood cell distribution width, blood urea nitrogen, direct bilirubin and low albumin were associated with severe COVID-19 infection (AUC 0.912, 95% CI 0.846–0.978), sensitivity: 85.71%, specificity: 87.58%. Similarly, Huang [38] found that hypoalbuminemia can predict the prognosis of COVID-19 and is not related to age and complications. Multivariate regression analysis showed that hypoalbuminemia was an independent risk factor for death (OR 6.394, 95% CI 1.315–31.092). Systematic review with meta-analysis showed that the increase of liver biochemistry during the first visit or illness is an important indicator of the severity of the disease. Low serum albumin indicates that this is a serious disease.The severity of elevated liver enzyme markers determines the outcome of COVID-19 [39]. Surprisingly, the incidence of liver injury is as high as 58–78% in the death cases of COVID-19 [40, 41]. In conclusion, there are many factors for poor prognosis of liver injury, which may be related to age (> 60 years old), severe COVID-19, basic diseases (hypertension, diabetes, cardiovascular disease, underlying liver diseases, etc.) and other related factors. If liver injury is combined with these factors, the patients may have a poor prognosis. Therefore, for spatients with severe COVID-19, require more in-depth monitoring or individualized treatment especially in elderly patients with underlying diseases or other complications.

## Management and treatment of liver injury

Studies have suggested that all COVID-19 patients should be regularly monitored for liver biochemical indicators. So far, data on the safety of drugs used to treat COVID-19 patients with liver injury are still missing, and treatment is mostly based on experience [20]. In a consensus statement of the American Association for the Study of Liver Diseases (AASLD) expert panel, it was stated that (1) etiologies unrelated to COVID-19, including other viruses such as hepatitis A, hepatitis B and hepatitis C, should be considered when evaluating patients with COVID-19 and elevated liver biochemical reactions. (2) Considering other causes of elevated liver biochemical responses, including myositis (especially glutamic oxalacetic transaminase > ALT), cardiac injury, ischemia, and cytokine release syndrome. (3) AST or ALT levels > 5 × ULN may result in the exclusion of such patients in some drug studies, but abnormal liver biochemical reactions should not be contraindicated in studies of COVID-19 or in super-indication treatments (e.g., Redsivir, Toxicizumab, chloroquine, hydroxychloroquine). (4) All hospitalized patients with COVID-19, especially those treated with redesivir or tocilizumab, should be regularly monitored for liver biochemical indicators regardless of baseline values. (5) In patients with autoimmune hepatitis or liver transplantation, patients with elevated COVID-19 activity and liver biochemical reactions should not be considered to have sudden onset of disease or acute cellular rejection without biopsy confirmation. (6) Patients with cirrhosis, autoimmune hepatitis treated with immunosuppressive drugs, and post-transplant patients receiving immunosuppressive therapy should be considered at increased risk for severe COVID-19 and should be prioritized for testing [42]. Other recommendations include that ongoing HBV and HCV antiviral therapy should be continued, but initiation of antiviral therapy in HCV patients may need to be delayed. Non-emergency patients may postpone liver ultrasonography or liver biopsy. Strict treatment indications should be followed when immunosuppressive agents are initiated in patients with liver disease, such as autoimmune hepatitis [AIH] or graft rejection. Immunosuppressants should be continued in patients with AIH or transplantation.

In addition, over the course of the pandemic, it is worth mentioning that liver transplantation (LT) patients should need special clinical management. Most institutions suggest that LT patients should delay the operation, except for critically ill patients [43]. Currently, there are insufficient data to show the relationship between immunosuppressive therapy and COVID-19 in LT recipients. However, here are two different opinions. From the perspective of the Beijing liver transplantation working group [44], LT recipients with mild or no infection with SARS-CoV-2 should continue to receive immunosuppressive therapy, but LT recipients with moderate to severe infection with SARS-CoV-2 should be given a reduced calcineurin inhibitor treatment dosage. Also, in order to reduce the severity of pneumonia, LT patientsinfected with COVID-19 should be given short-term steroid therapy. On the contrary, the position statement in EASL-ESCMID recommended [43] that the dose of immunosuppressant drugs can be adjusted according to antiviral treatment regimens, because it is likely that the drugs in both regimens will interact with each other. During the pandemic, for patients undergoing liver transplantation, SARS-CoV-2 should be detected to prevent infection.

## Conclusion

In conclusion, in COVID-19 disease, elevated liver enzymes are usually mild and generally recover without treatment. In clinical practice, we need to distinguish whether the onset of abnormal liver function occurs at diagnosis or during treatment.

## Data Availability

Not applicable.

## Abbreviations

ALT: Alanine amino transaminase
AST: Aspartate amino transaminase
TBIL: Total bilirubin
GGT: γ-Glutamyl transpeptidase
ACE2: Angiotensin-converting enzyme 2
CoV: Coronaviruses
COVID-19: Coronavirus disease 2019
ICU: Intensive care unit
SARS-CoV-2: Severe Acute Respiratory Syndrome Coronavirus-2
USA: United States of America
WHO: World Health Organization
ULN: Upper limits of normal
HBV: Hepatitis B virus
HCV: Hepatitis C virus
LT: Liver transplantation
AIH: Autoimmune hepatitis
